# Alteration in Intracellular Zn^2+^ Homeostasis as a Result of TRPM2 Channel Activation Contributes to ROS-Induced Hippocampal Neuronal Death

**DOI:** 10.3389/fnmol.2017.00414

**Published:** 2017-12-12

**Authors:** Xin Li, Wei Yang, Lin-Hua Jiang

**Affiliations:** ^1^School of Biomedical Sciences, Faculty of Biological Sciences, University of Leeds, Leeds, United Kingdom; ^2^Department of Neurobiology, School of Medicine, Zhejiang University, Hangzhou, China; ^3^Sino-UK Joint Laboratory of Brain Function and Injury of Henan Province and Department of Physiology and Neurobiology, Xinxiang Medical University, Xinxiang, China

**Keywords:** TRPM2 channel, hippocampal neuronal death, ROS, intracellular Zn^2+^ homeostasis, lysosomal dysfunction, mitochondrial dysfunction

## Abstract

Transient receptor potential melastatin-related 2 (TRPM2) channel, a molecular sensor for reactive oxygen species (ROS), plays an important role in cognitive dysfunction associated with post-ischemia brain damage thought to result from ROS-induced TRPM2-dependent neuronal death during reperfusion. Emerging evidence further suggests that an alteration in the Zn^2+^ homeostasis is critical in ROS-induced TRPM2-dependent neuronal death. Here we applied genetic and pharmacological interventions to define the role of TRPM2 channel in ROS-induced neuronal death and explore the mechanisms contributing in the alteration in intracellular Zn^2+^ homeostasis in mouse hippocampal neurons. Exposure of neurons to 30–300 μM H_2_O_2_ for 2–24 h caused concentration/duration-dependent neuronal death, which was significantly suppressed, but not completely prevented, by TRPM2-knockout (TRPM2-KO) and pharmacological inhibition of the TRPM2 channel. H_2_O_2_-induced neuronal death was also attenuated by treatment with TPEN acting as a Zn^2+^ selective chelator. Single cell imaging demonstrated that H_2_O_2_ evoked a prominent increase in the intracellular Zn^2+^ concentration, which was completely prevented by TPEN as well as TRPM2-KO and inhibition of the TRPM2 channel. Furthermore, H_2_O_2_ induced lysosomal Zn^2+^ release and lysosomal dysfunction, and subsequent mitochondrial Zn^2+^ accumulation that provokes mitochondrial dysfunction and ROS generation. These H_2_O_2_-induced lysosomal/mitochondrial effects were prevented by TRPM2-KO or TPEN. Taken together, our results provide evidence to show that a dynamic alteration in the intracellular Zn^2+^ homeostasis as a result of activation of the TRPM2 channel contributes to ROS-induced hippocampal neuronal death.

## Introduction

Brain is highly demanding for metabolism and energy and thus is prone to damage by ischemia, if it lasts long or becomes severe, and also by reperfusion after transient ischemia (McCord, 1985; Kalyanaraman, 2013). Hippocampus is crucial for brain functions such as learning and memory, and studies of both animal models of ischemia-reperfusion and ischemic stroke patients have documented that hippocampal neuronal death during reperfusion plays an important role in progressive decline in the cognitive function after ischemia stroke (Kitagawa et al., 1990; Doyle et al., 2008). Reperfusion-induced brain damage is strongly and causatively related to generation of excessive reactive oxygen species (ROS) upon re-introduction of oxygen molecule during reperfusion (McCord, 1985; Chan, 2001; Uttara et al., 2009; Chen et al., 2011; Sanderson et al., 2013). To gain a better understanding of ROS-induced neuronal death is therefore critical in developing therapeutics targeting reperfusion-related neuronal death, which is currently non-existent, to ameliorate post-ischemia cognitive dysfunction.

It is known that ROS cause DNA damage and the poly(ADP-ribose) polymerase-1 (PARP-1) activity is critical in the process of repairing DNA damage (Dantzer et al., 2006). However, it is also well recognized that prolonged activation or hyper-activation of PARP-1 induces cell death, including neurotoxicity (Cole and Perez-Polo, 2002; Lo et al., 2003; Alano et al., 2010; Virág et al., 2013). ADP-ribose (ADPR), long known as a biochemical by-product of the PARP-1-dependent DNA repair process, has been relatively recently established as an intracellular signaling molecule due to its capacity of selectively gating the transient receptor potential melastatin-related 2 (TRPM2) Ca^2+^-permeable cationic channel (Perraud et al., 2001; Sano et al., 2001). An early study showed that activation of the TRPM2 channel mediates ROS-induced cell death (Hara et al., 2002) and recent studies using transgenic TRPM2-knockout (TRPM2-KO) mice have revealed a critical role for ROS-induced TRPM2-mediated cell death in diverse cell types that contributes to pathologies such as diabetes (Manna et al., 2015), ischemic kidney damage (Gao et al., 2014) and paracetamol-induced liver injury (Kheradpezhouh et al., 2014). In the brain, it has been shown that the TRPM2 channel is expressed in hippocampal (Olah et al., 2009; Verma et al., 2012; Ye et al., 2014), cortical (Kaneko et al., 2006), striatal (Fonfria et al., 2005; Kaneko et al., 2006) and dopaminergic neurons (Sun et al., 2016) as well as microglial cells (Kraft et al., 2004; Fonfria et al., 2006; Mortadza et al., 2017), neurovascular endothelial cells (Park et al., 2014) and pericytes (Jiang et al., 2017). Previous studies support a role for the TRPM2 channel, particularly TRPM2-mediated increase in the cytosolic Ca^2+^ concentration ([Ca^2+^]_c_), in ROS-induced neuronal death (Fonfria et al., 2005; Kaneko et al., 2006; Verma et al., 2012). Furthermore, TRPM2-dependent neuronal death has been related to post-ischemia brain damage (Jia et al., 2011; Nakayama et al., 2013; Shimizu et al., 2013, 2016; Ye et al., 2014) and Alzheimer’s disease (Ostapchenko et al., 2015). The molecular mechanisms underlying ROS-induced TRPM2-dependent neuronal death however remain less well-defined.

Zn^2+^ is a trace metal ion that is biologically important, serving an essential enzyme cofactor and transcription factor, but Zn^2+^ is also well-known for being neurotoxic. In agreement with the finding from a recent study that the TRPM2 channel selectively mediates brain damage induced by reperfusion, not by ischemia (Alim et al., 2013), our recent study has revealed an exclusive role for the TRPM2 channel in sustaining the cytosolic Zn^2+^ concentration ([Zn^2+^]_c_) during reperfusion that is causatively associated with post-ischemia neuronal death (Ye et al., 2014), implying TRPM2-dependent rise in the [Zn^2+^]_c_ is critical in driving ROS-induced neuronal death. As has been well recognized, Zn^2+^-induced neuronal death results in significant part from the potency of cytosolic Zn^2+^ in inducing lysosomal and mitochondrial dysfunction (Jiang et al., 2001; Dineley et al., 2003, 2005; Hwang et al., 2008; Medvedeva et al., 2009; Sensi et al., 2011; Shuttleworth and Weiss, 2011). Therefore, in the present study, we performed experiments using cultured hippocampal neurons to investigate the contribution of TRPM2 channel, particularly TRPM2-dependent alterations in the intracellular Zn^2+^ homeostasis, lysosomal function and mitochondrial function, in ROS-induced neuronal death.

## Materials and Methods

### Chemicals and Reagents

General chemicals and reagents used in the study were obtained from Sigma, except those indicated specifically. PJ34 was from Calbiochem, and TPEN from StressMarq Biosciences.

### Preparation of Primary Hippocampal Neurons

All experiments and experimental protocols, including all those involving mice, were approved by the University of Leeds Ethical Review Committee and performed in accordance with the University of Leeds guidelines and procedure and conforming to the UK Home Office rules and regulations. The TRPM2-KO mice were generated in our previous study (Zou et al., 2013). Primary cultures of hippocampal neurons from postnatal 1–2 day old C57BL/6 mice of both sex were prepared according to the protocols previously described (Beaudoin et al., 2012). In brief, hippocampal tissues were collected into ice-cold Hank’s balanced salt solution (Invitrogen) and kept in <30 min before incubated in 0.125% trypsin-EDTA solution (Life Technologies) in 37°C for 15 min, and then in DMEM/F12 containing 10% horse serum (Thermo Scientific). The tissues were triturated by pipetting and filtered through a 70-μm nylon cell strainer (Fisher Scientific) into a 50-ml Falcon tube to obtain single cell suspension. Cells were collected by centrifugation at approximately 100 *g* for 5 min, and re-suspended in fresh DMEM/F12 medium supplemented with 10% horse serum, 5 units/ml penicillin and 50 μg/ml streptomycin. Cells were seeded at 100 cells/mm^2^ and cultured in the above culture medium for 4 h before maintained in Neurobasal medium with 2% serum-free B27 supplement (Thermo Scientific), 0.5 mM L-glutamine, 5 units/ml penicillin and 50 μg/ml streptomycin. After 2 days, cytosine β-D-arabinofuranoside was added with a final concentration of 0.5 μM to inhibit microglia growth. The medium was changed twice a week. Neurons cultured for 14–16 days *in vitro* were used.

### Measurement of Neuronal Death

Neuronal death was determined using propidium iodide (PI) staining as previously described with slight modifications (Xu et al., 2012). Neurons were seeded in 24-well poly-L-lysine-coated plates (Sarstedt) and treated with H_2_O_2_ at indicated concentrations and durations. In some experiments, culturing medium was added with 5 mM EGTA to examine the effect of removing extracellular Ca^2+^ on H_2_O_2_-induced neuronal death. Inhibitors at indicated concentrations were added 30 min before and during exposure to H_2_O_2_. Neurons were incubated with 1 μg/ml PI and 1 μM Hoechst 33342 (Cell Signaling Technology) for 30 min immediately after treatment with H_2_O_2_. Images were captured using an EVOS^®^ Cell Imaging System (Life Technologies), and ImageJ was used for cell counting.

### Immunocytochemistry

Neurons were seeded on a 13-mm poly-L-lysine-coated coverslips placed in a 24-well plate. After rinsed with phosphate buffer saline (PBS), neurons were incubated in Zamboni’s fixative (15% (v/v) picric acid and 5.5% (v/v) formaldehyde in PBS) for 1 h, rinsed with PBS, and incubated with blocking serum solution (10% (v/v) goat serum and 4% (v/v) Triton X-100 in PBS) for 1 h. Neurons were incubated with primary rabbit anti-TRPM2 (1:1000; Bethyl) or mouse anti-Cyt-c antibody (1:100; BD Pharmingen) overnight at 4°C and, after washing in PBS, with secondary anti-rabbit or anti-mouse antibody conjugated with fluorescein isothiocyanate (1:500) for 1 h. Neurons were washed with PBS and rinsed in water before mounted with SlowFade^®^ Gold Antifade (Invitrogen) and kept in 4°C. Images were captured using an inverted Zeiss LSM880 confocal microscope with a 63× objective. ImageJ was used for analysis of Cyt-c immunostaining.

### Single Live Cell Confocal Imaging

Neurons were seeded in 35-mm poly-L-lysine-coated glass bottom dishes (World Precision Instruments) 24 h before use. After the medium was removed, neurons were rinsed with standard buffer solution (SBS: 130 mM NaCl, 1.5 mM CaCl_2_, 5 mM KCl, 1.2 mM MgCl_2_, 8 mM glucose, 10 mM HEPES, pH 7.4). For Zn^2+^ imaging, neurons were incubated in SBS containing 1 μM FluoZin3-AM (Life Technologies) and 0.01% (w/v) pluronic acid for 1 h. In some experiments, 25 nM MitoTracker Red CMXRos or 1 μM LysoTracker Red DND-99 (Life Technologies) was also included. For characterization of mitochondrial morphology, neurons were incubated with 100 nM MitoTracker Green FM (Life Technologies) at 37°C for 30 min. Neurons were rinsed with SBS and maintained in 2 ml PBS before images were captured using an inverted Zeiss LSM880 microscope with a 63× objective. For time-lapse confocal imaging, neurons in petri-dishes were mounted on the scanning stage of confocal microscope and an area was randomly chosen for recording for 30 min with images captured every 5 min. ImageJ was used for analysis of the fluorescence intensity.

### Measurement of Production of Mitochondrial ROS

Production of mitochondrial ROS (MitoROS) was determined using MitoTracker Red CM-H_2_Xros according to the manufacturer’s instructions (Life Technologies). Briefly, after indicated treatments, neurons were incubated in culture medium containing 100 nM MitoTracker Red CM-H_2_Xros for 30 min at 37°C. Medium were replaced with PBS before images were captured using an EVOS^®^ Cell Imaging System. ImageJ was used for analysis of the fluorescence intensity.

### Data Presentation and Statistical Analysis

Neuronal death was presented by expressing the number of PI positive cells as percentage of all cells identified by Hoechst staining in the same areas. Co-localization of two fluorescence signals was quantified by Pearson’s correlation coefficient as detailed in a previous study (Dunn et al., 2011), with its value varying between −1 and 1 that represent total negative and total positive correlation, respectively. The mitochondria morphology was characterized by computer-assisted analysis of the aspect ratio (major axis/minor axis) and form factor (reciprocal of circularity value), as described in previous studies (De Vos et al., 2005; Koopman et al., 2005). Data are presented, where appropriately, as mean ± SEM. Statistical significance analysis was conducted using one-way ANOVA with *post hoc* Tukey’s test, with significance at the level of *p* < 0.05.

## Results

### TRPM2 Channel and Zn^2+^ Are Involved in H_2_O_2_-Induced Hippocampal Neuronal Death

**Figure 1:**
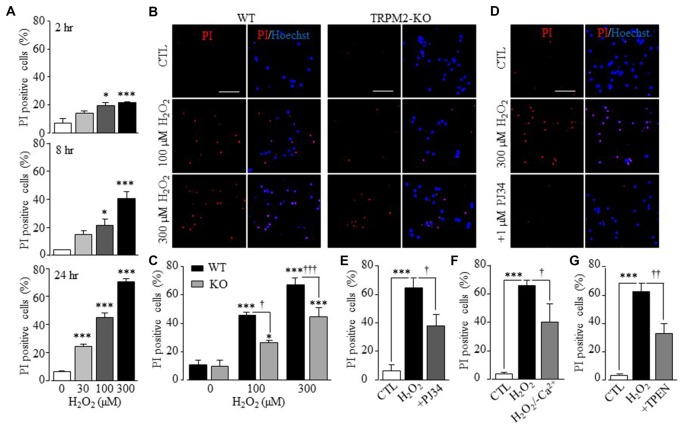
Transient receptor potential melastatin-related 2 (TRPM2) has a significant role in H_2_O_2_-induced hippocampal neuronal death.** (A)** Mean percentage of propidium iodide (PI) positive WT neurons after treatment with H_2_O_2_ for indicated conditions, from 3 to 8 independent experiments with each independent experiment examining 350–500 neurons. *p < 0.05 and ***p < 0.005 indicate difference from control. **(B)** Representative images showing PI and Hoechst staining of WT and TRPM2-KO neurons treated with indicated concentrations of H_2_O_2_ for 24 h. **(C)** Mean percentage of PI positive neurons under indicated conditions as shown in panel **(B)**, from 4 to 5 independent experiments with each experiment examining 350–550 neurons. *p < 0.05 and ***p < 0.005 indicate difference from respective untreated neurons. ^†^*p* < 0.05 and ^†††^*p* < 0.005 indicate difference between WT and TRPM2-KO neurons under the same treatments. **(D)** Representative images showing PI and Hoechst staining of WT neurons treated with 1 μM PJ34, 30 min prior to and during exposure to 300 μM H_2_O_2_ for 24 h. **(E–G)** Mean percentage of PI positive neurons under indicated conditions, from 4 to 5 independent experiments with each experiment examining 350–550 neurons. ***p < 0.005 indicates difference from control. ^†^*p* < 0.05 and ^††^*p* < 0.01 indicate difference from neurons exposed to H_2_O_2_ alone. Scale bar is 100 μm in **(B,D)**.

Previous studies examining cortical, striatal and hippocampal neuronal preparations demonstrated involvement of the TRPM2 channel in ROS-induced neuronal death (Fonfria et al., 2005; Kaneko et al., 2006; Verma et al., 2012), but the exact contribution of TRPM2 channel in such neuronal death was not well-defined. We therefore started with examining neuronal death in hippocampal neurons prepared from WT and TRPM2-KO mice, using PI staining. Exposing neurons to H_2_O_2_ at 30–300 μM for 2–24 h induced concentration- and duration-dependent neuronal death in WT neurons (Figure 1A and Supplementary Figure S1). H_2_O_2_-induced neuronal death was significantly attenuated but not completely prevented by TRPM2-KO (Figures 1B,C). Consistently with critical involvement of PARP-1 in ROS-induced TRPM2 channel activation (Jiang et al., 2010), treatment with PJ34, a PARP-1 inhibitor, before and during exposure to H_2_O_2_ significantly attenuated H_2_O_2_-induced neuronal death in WT neurons, but not in TRPM2-KO neurons (Figures 1D,E and Supplementary Figure S2). Taken together, these genetic and pharmacological results provide unambiguous evidence to show that the TRPM2 channel plays a significant but not exclusive role in ROS-induced neuronal death. Removal of extracellular Ca^2+^ reduced H_2_O_2_-induced neuronal death (Figure 1F), suggesting TRPM2-mediated Ca^2+^ influx is involved in neuronal death, as previously reported for H_2_O_2_-induced cortical neuronal death (Kaneko et al., 2006). Furthermore, H_2_O_2_-induced neuronal death was also markedly inhibited by treatment with TPEN at 1 μM, which acts as a selective Zn^2+^ chelator, prior to and during exposure to H_2_O_2_ (Figure 1G), indicating a critical role for Zn^2+^ in H_2_O_2_-induced neuronal death.

### TRPM2 Channel in H_2_O_2_-Induced Increase in the [Zn^2+^]_c_ and Lysosomal Dysfunction

**Figure 2:**
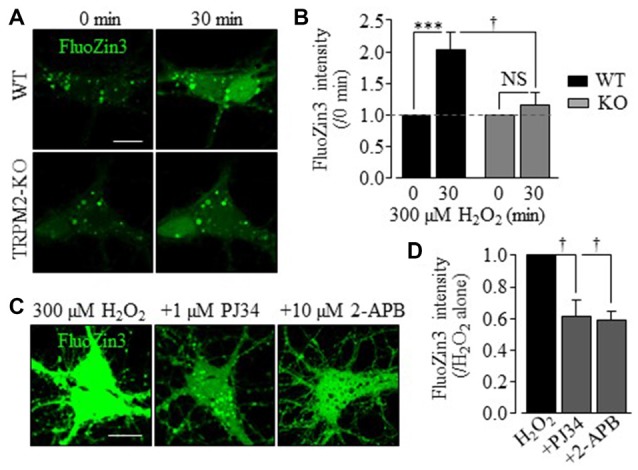
TRPM2 is critical in H_2_O_2_-induced increase in the [Zn^2+^]_c_ in hippocampal neurons. **(A)** Representative time-lapse confocal images showing FluoZin3 (green) fluorescence in WT (top two panels) and TRPM2-KO (bottom two panels) neurons before and after exposed to 300 μM H_2_O_2_ for 30 min. **(B)** Mean FluoZin3 fluorescence intensity under indicated conditions from 9 to 11 independent experiments with a total of 40 neurons examined. All values are normalized to the basal fluorescence level in matched experiments. ***p < 0.005 indicates difference from the basal level and ^†^*p* < 0.05 for difference between WT and TRPM2-KO neurons treated with H_2_O_2_. NS, no significant difference. **(C)** Representative confocal images showing FluoZin3 fluorescence in WT neurons treated with 300 μM H_2_O_2_ for 30 min or treated with 1 μM PJ34 or 10 μM 2-APB, 30 min prior to and during exposure to H_2_O_2_. **(D)** Mean FluoZin3 fluorescence intensity in neurons under indicated conditions, expressed relative to the fluorescence level in neurons exposed to 300 μM H_2_O_2_ alone, from four to five independent experiments with each experiment examining 20–25 neurons. ^†^*p* < 0.05 indicates difference from neurons treated with H_2_O_2_ alone. Scale bar is 10 μm in **(A,C)**.

**Figure 3:**
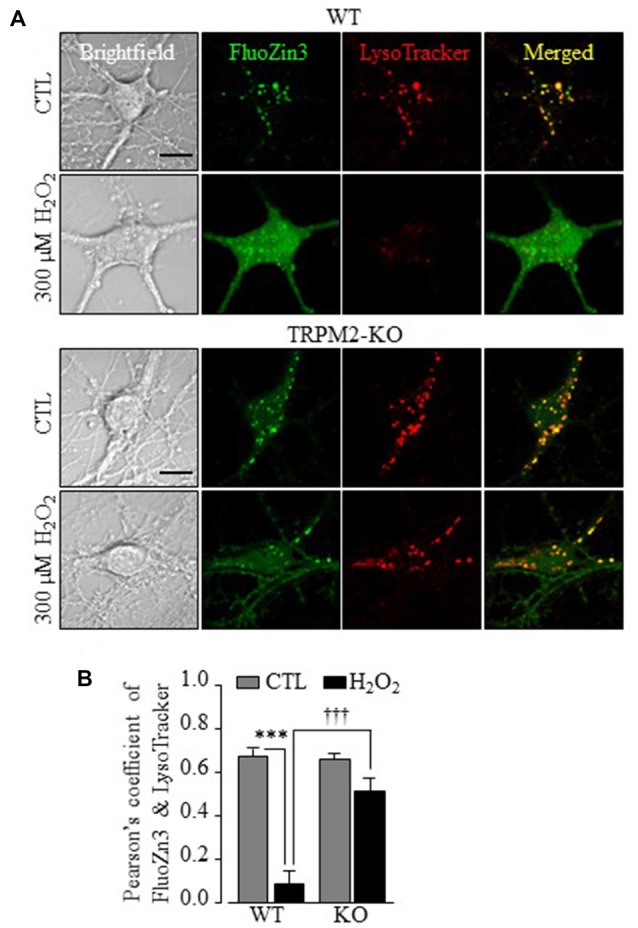
TRPM2 is required in H_2_O_2_-induced lysosomal dysfunction, cytosolic Zn^2+^ increase in hippocampal neurons.** (A)** Representative confocal images showing FluoZin3 (green) and LysoTracker (red) in WT or TRPM2-KO neurons under control (CTL) or treatment with 300 μM H_2_O_2_ for 30 min. Scale bar is 10 μm. **(B)** Mean Pearson’s correlation coefficient from three independent experiments with each experiment analyzing 12–18 neurons. ***p < 0.005 indicates difference from control and ^†††^*p* < 0.005 indicates difference between WT and TRPM2-KO neurons treated with H_2_O_2_.

To better understand the role of Zn^2+^ in H_2_O_2_-induced neuronal death, particularly its relationships to the TRPM2 channel activation, we performed single live cell confocal imaging using FluoZin3, a fluorescent Zn^2+^ indicator (Gee et al., 2002) to examine the [Zn^2+^]_c_. There was a very low but discernible level of free Zn^2+^ that was predominantly present in puncta in untreated hippocampal neurons (Figures 2A, 3A), and exposure to H_2_O_2_ for 30 min induced a salient increase in the [Zn^2+^]_c_ in WT neurons, which was almost completely absent in TRPM2-KO neurons (Figures 2A,B), as reported in our recent study (Ye et al., 2014). Furthermore, treatment with PJ34, or 2-APB, a TRPM2 channel blocker, significantly reduced H_2_O_2_-induced increase in the [Zn^2+^]_c_ in WT neurons (Figures 2C,D). Treatments with these agents did not significantly alter the Zn^2+^ puncta and [Zn^2+^]_c_ in TRPM2-KO neurons (Supplementary Figure S3). These results strongly support that the TRPM2 channel activation is required for H_2_O_2_-induced increase in the [Zn^2+^]_c_.

To provide insights into TRPM2-dependent alteration in the intracellular Zn^2+^ homeostasis, we carried out further experiments using FluoZin3 in combination with intracellular organelle specific fluorescence markers. In untreated neurons, a majority of the Zn^2+^ puncta exhibited strong correlation with LysoTracker, which was similar in WT and TRPM2-KO neurons (Figures 3A,B), suggesting that they are primarily located in lysosomes and not altered by TRPM2-KO. Exposure of WT neurons to H_2_O_2_ induced substantial loss of LysoTracker fluorescence in addition to an increase in the [Zn^2+^]_c_ (Figure 3A), suggesting lysosomal dysfunction. These effects were largely ablated in TRPM2-KO neurons (Figures 3A,B), indicating strong dependence of H_2_O_2_-induced lysosomal dysfunction on the TRPM2 channel.

### H_2_O_2_ Induces TRPM2-Dependent Mitochondrial Zn^2+^ Accumulation, Dysfunction and ROS Generation

**Figure 4:**
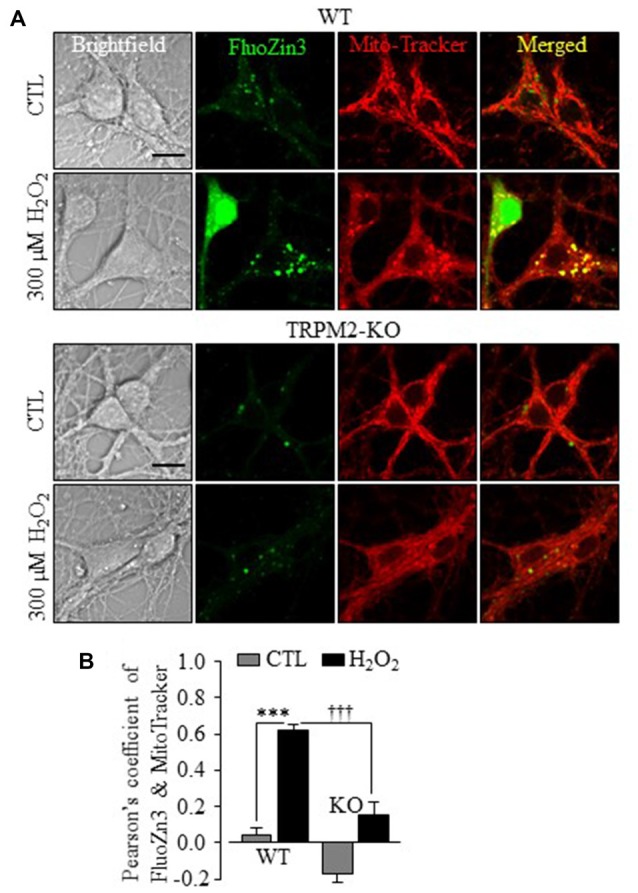
TRPM2 channel plays a critical role in H_2_O_2_-induced mitochondrial Zn^2+^ accumulation in hippocampal neurons.** (A)** Representative confocal images showing FluoZin3 (green) and MitoTracker (red) in WT or TRPM2-KO neurons under control (CTL) or treatment with 300 μM H_2_O_2_ for 30 min. Scale bar is 10 μm. **(B)** Mean Pearson’s correlation coefficient from three independent experiments with each experiment analyzing 12–18 neurons. ***p < 0.005 indicates difference from control, and ^†††^*p* < 0.005 indicates difference between WT and TRPM2-KO neurons treated with H_2_O_2_.

**Figure 5:**
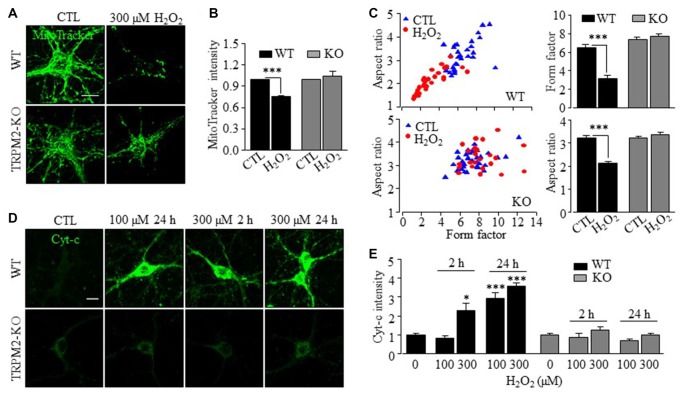
TRPM2 channel has a critical role in H_2_O_2_-induced mitochondrial loss, mitochondrial fragmentation and cytochrome-c release in hippocampal neurons.** (A)** Representative confocal images showing MitoTracker Green fluorescence in WT (top two panels) or TRPM2-KO (bottom two panels) neurons under control (CTL) or treatment with 300 μM H_2_O_2_ for 30 min. **(B)** Mean MitoTracker Green fluorescent intensity in WT and TRPM2-KO neurons under indicated condition from 3 independent experiments with 10–12 neurons examined in each experiment. All values were normalized to control neurons in matched experiments. ***p < 0.005 indicates difference from control. **(C)** Computer-assisted analyses of mitochondria morphology. *Left*, distribution of the factor and aspect ratio values of mitochondria in neurons from 3 independent experiments with 10–12 neurons examined in each experiment. Blue triangles and red circles represent control neurons and neurons treated with 300 μM H_2_O_2_ for 30 min, respectively. *Right*, mean values of the form factor (top) and aspect ratio (bottom). ***p < 0.005 indicates difference from control. **(D)** Immunofluorescent images showing cytochrome-c (Cyt-c) staining in WT (top panels) and TRPM2-KO (bottom panels) neurons under indicated conditions. **(E)** Mean Cyt-c staining fluorescence intensity from three to four independent experiments with 15–25 neurons examined in each experiment. The values were presented relative to control neurons in matched experiments. *p < 0.05 and ***p < 0.005 indicate difference from control. Scale bar is 10 μm in **(A,D)**.

By contrast with LysoTracker, there was little correlation between the Zn^2+^ puncta and MitoTracker in WT and TRPM2-KO neurons under control conditions (Figures 4A,B). Exposure to H_2_O_2_ induced a noticeable increase in the co-localization of FluoZin3 and MitoTracker fluorescence (Figures 4A,B), suggesting mitochondrial Zn^2+^ accumulation. However, such mitochondrial Zn^2+^ accumulation was not observed in TRPM2-KO neurons (Figures 4A,B). Exposure to H_2_O_2_ resulted in a significant reduction in the MitoTracker fluorescence intensity in WT neurons (Figures 5A,B), indicative of mitochondrial dysfunction. In addition, mitochondria exhibited the typical tubular morphology in untreated WT neurons and became noticeably fragmented in neurons after being exposed to H_2_O_2_ (Figure 5A). Such changes were more clearly shown by quantitative analysis of the form factor and aspect ratio (Figure 5C), two geometric parameters widely used to characterize mitochondrial morphology (see “Materials and Methods” section). Furthermore, H_2_O_2_ induced mitochondrial release of cytochrome c (Cyt-c) that became accessible to antibody labeling (Figures 5D,E). H_2_O_2_-induced reduction in the MitoTracker fluorescence intensity, mitochondrial fragmentation and Cyt-c release were almost completely abrogated by TRPM2-KO (Figures 5A–E), demonstrating critical dependence of H_2_O_2_-induced mitochondrial dysfunction on the TRPM2 channel.

**Figure 6:**
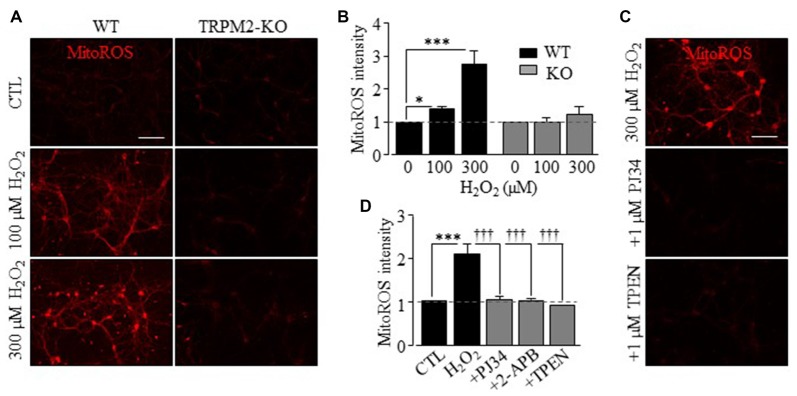
TRPM2 channel is crucial for H_2_O_2_-induced production of mitochondrial reactive oxygen species (ROS) in hippocampal neurons.** (A,C)** Representative fluorescence images showing MitoTracker Red CM-H_2_Xros (MitoROS) fluorescence in WT (left column) or TRPM2-KO (right column) neurons under control (CTL) and treatment with 100 μM or 300 μM H_2_O_2_ for 2 h **(A)**, or in WT neurons treated with 1 μM PJ34 or 1 μM TPEN, 30 min before and during exposure to 300 μM H_2_O_2_ **(C)**. Scale bar is 100 μm. **(B,D)** Mean MitoROS fluorescence intensity from 3 to 5 independent experiments with 35–70 neurons examined in each experiment, presented relative to the fluorescence level in control neurons in matched experiments. 2-APB was used at 100 μM. *p < 0.05 and ***p < 0.005 indicate significant difference from control. ^†††^*p* < 0.005 indicates significant difference from neurons exposed to 300 μM H_2_O_2_ alone.

We finally examined whether mitochondrial Zn^2+^ accumulation stimulated production of mitochondrial ROS in hippocampal neurons, using MitoTracker Red CM-H_2_Xros (MitoROS), a fluorescent indicator of mitochondrial ROS. Exposure to H_2_O_2_ resulted in a strong increase in MitoROS fluorescence in WT neurons, indicating production of mitochondrial ROS. Such production of mitochondrial ROS was not observed in TRPM2-KO neurons (Figures 6A,B) or in WT neurons after treatment with PJ34 or 2-APB (Figures 6C,D), supporting a critical role for the TRPM2 channel activation in production of mitochondrial ROS. Furthermore, H_2_O_2_-induced production of mitochondrial ROS was also inhibited by treatment with TPEN, indicating a causative relationship with mitochondrial Zn^2+^ accumulation with production of mitochondrial ROS (Figures 6C,D). Taken together, these results provide compelling evidence to show a key role for the TRPM2 channel in H_2_O_2_-induced mitochondrial Zn^2+^ accumulation that induces production of mitochondrial ROS.

## Discussion

Here we provide evidence to show that a significant but not exclusive role of the TRPM2 channel in ROS-induced neuronal death and reveal TRPM2-dependent dynamic alterations in the intracellular Zn^2+^ homeostasis, lysosomal and mitochondrial functions that are important in ROS-induced neuronal death.

Previous studies using cortical, striatal and hippocampal neurons (Fonfria et al., 2005; Kaneko et al., 2006; Verma et al., 2012) support a role for the TRPM2 channel in H_2_O_2_-induced neuronal death, but the exact contribution and the underlying molecular mechanisms remained not well-defined. Here we presented genetic and pharmacological results to confirm the previous findings (Figure 1A and Supplementary Figures S1, S2). More specifically, our results indicate that the TRPM2 channel has a significant but not exclusive role in mediating ROS-induced neuronal death. Furthermore, we provide several lines of evidence to support that TRPM2-dependent alteration in the intracellular Zn^2+^ homeostasis is critical in ROS-induced hippocampal neuronal death. First of all, H_2_O_2_-induced neuronal death was strongly inhibited by TPEN at a concentration that specifically acts as a Zn^2+^ chelator (Figure 1F). Second, single live cell imaging showed that exposure to H_2_O_2_ for 30 min evoked a prominent increase in the [Zn^2+^]_c_ (Figure 2) or alteration in the intracellular Zn^2+^ homeostasis in neurons (Figures 3, 4). Immunostaining showed that such exposure to H_2_O_2_ resulted in no discernible effect on the overall expression of TRPM2 protein, but appeared to slightly change its subcellular location (Supplementary Figure S4), and further study may help to clarify the implication of such a change. It is well recognized that an increase in the [Zn^2+^]_c_, as a result from extracellular Zn^2+^ influx or internal Zn^2+^ release through Zn^2+^-specific transporters as well as diverse Ca^2+^-transporting mechanisms, can induce neuronal death (Colvin et al., 2003; Medvedeva et al., 2009; Sensi et al., 2011; Shuttleworth and Weiss, 2011; Li et al., 2015). In the present study, we demonstrated a critical role for the TRPM2 channel in ROS-induced increase in the [Zn^2+^]_c_ (Figure 2 and Supplementary Figure S3) or alteration in the intracellular Zn^2+^ homeostasis (Figures 3, 4). Finally, inhibition of such Zn^2+^ signaling significantly attenuated ROS-induced neuronal death (Figure 1F). Considered that reperfusion stimulates generation of excessive ROS (McCord, 1985; Kalyanaraman, 2013), the findings from the present *in vitro* study, together with those from our recent *in vivo* study (Ye et al., 2014), consistently supports the notion that an alteration in the intracellular Zn^2+^ homeostasis via activation of the TRPM2 channel contributes to ROS-induced and reperfusion-related neuronal death.

We showed in the present study that H_2_O_2_-induced hippocampal neuronal death was also attenuated by removing extracellular Ca^2+^ (Figure 1E), as reported in a previous study for H_2_O_2_-induced cortical neuronal death (Kaneko et al., 2006). These findings suggests TRPM2-mediated Ca^2+^ influx is critical in determining H_2_O_2_-induced neuronal death. It is well recognized that intracellular Ca^2+^ is important for full activation of the TRPM2 channel by ADPR (Du et al., 2009; Tóth and Csanády, 2010). Our recent study shows functional expression of the TRPM2 channel on the cell surface, particularly requirement of extracellular Ca^2+^ influx for H_2_O_2_-induced increase in the [Zn^2+^]_c_ in hippocampal neurons (Ye et al., 2014). Thus, one explanation, which can readily reconcile the role of the [Ca^2+^]_c_ and [Zn^2+^]_c_ in ROS-induced neuronal death, is that TRPM2-mediated Ca^2+^ influx promotes further activation of the TRPM2 channel that leads to TRPM2-dependent alteration in the intracellular Zn^2+^ homeostasis, as recently proposed by studies examining the role of TRPM2 channel in H_2_O_2_-induced pancreatic β-cell death (Manna et al., 2015).

In the present study, we have provided new insights into TRPM2-dependent alteration in the intracellular Zn^2+^ homeostasis leading to ROS-induced neuronal death. As previously reported in cortical (Colvin et al., 2003) and hippocampal neurons (Ye et al., 2014), the present study revealed that hippocampal neurons under control conditions contained a very low level of Zn^2+^ that was largely present in puncta (Figure 2), and further demonstrated that a majority of such Zn^2+^ puncta were strongly co-localized with LysoTracker (Figures 3A,B), suggesting lysosomal localization. Similar findings were described in insulin-secreting cells (Manna et al., 2015). Moreover, H_2_O_2_-induced increase in the [Zn^2+^]_c_ in hippocampal neurons was accompanied with lysosome dysfunction as evidenced by the massive reduction in LysoTracker fluorescence intensity (Figure 3A). Genetic deletion of the TRPM2 channel not only prevented H_2_O_2_-induced increase in the [Zn^2+^]_c_ (Figures 2A,B) but also H_2_O_2_-induced lysosomal dysfunction (Figure 3A), leading us to propose that lysosomal dysfunction is at least in part responsible for lysosomal Zn^2+^ release to increase the [Zn^2+^]_c_. However, other Zn^2+^ release mechanisms and sources remain possible. For example, the TRPM2 channel was shown to function as a lysosomal Ca^2+^ release channel in insulin-secreting cells (Togashi et al., 2006; Lange et al., 2009) and has been recently suggested to mediate lysosomal Zn^2+^ release that is responsible for ROS-induced increase in the [Zn^2+^]_c_ and subsequent pancreatic-β cell death (Manna et al., 2015). Further studies are required to examine whether a similar mechanism operates in hippocampal neurons. It is noteworthy to mention that increasing indirect evidence supports a role for the TRPM2 channel in modulating intracellular Zn^2+^ homeostasis (Ye et al., 2014; Manna et al., 2015; Abuarab et al., 2017; Li et al., 2017). However, unlike the Ca^2+^-permeability (Xia et al., 2008), it remains difficult to demonstrate whether the TRPM2 channel is permeable to Zn^2+^ because of the potent inhibition of TRPM2 channel by micromolar concentrations of Zn^2+^ (Yang et al., 2011). There is evidence that ROS induces Zn^2+^ release from cytosolic Zn^2+^-binding proteins such as metallothioneins in hippocampal neurons (Lee et al., 2003), which cannot be ruled out as a possible source for H_2_O_2_-induced increase in the [Zn^2+^]_c_ observed in the present study.

In this study, we also provide evidence to suggest that mitochondrial Zn^2+^ accumulation and ensuring mitochondrial ROS generation are important in ROS-induced hippocampal neuronal death. The Zn^2+^ puncta in untreated neurons were poorly co-localized with MitoTracker, but there was a considerable increase in the col-localization between Zn^2+^ and MitoTracker in H_2_O_2_-treated neurons, suggesting mitochondrial Zn^2+^ accumulation (Figure 4). H_2_O_2_-induced Zn^2+^ translocation into mitochondria was however not observed in TRPM2-KO neurons (Figure 4). Furthermore, H_2_O_2_ induced mitochondrial dysfunction (Figures 5A,B), mitochondrial fragmentation (Figures 5A,C) and release of Cyt-c (Figures 5D,E). Similar Zn^2+^-induced mitochondrial effects were previously reported in cortical neurons (Jiang et al., 2001; Dineley et al., 2003; Sensi et al., 2003, 2011; Medvedeva et al., 2009; Shuttleworth and Weiss, 2011). Here we provide the first evidence to show that these H_2_O_2_-induced mitochondrial effects in hippocampal neurons were strongly dependent of the TRPM2 channel (Figure 5). Of notice, a recent study reports that genetic depletion of the TRPM2 expression in human neuroblastoma SH-SY5Y cells led to an increased mitochondrial ROS level as well as reduced cell proliferation (Bao et al., 2016), although it was unclear regarding the implication to ROS-induced cell death. Nonetheless, another recent study shows that activation of the TRPM2 channel mediates SH-SY5Y cell death induced by H_2_O_2_ as well as 1-methyl-4-phenylpyridinium ion (Sun et al., 2016). We found negligible production of mitochondrial ROS in both WT and TRPM2-KO neurons under control conditions (Figures 6A,B). However, H_2_O_2_ induced substantial production of mitochondrial ROS in hippocampal neurons that was prevented by genetic deletion or pharmacological inhibition of the TRPM2 channel (Figures 6A–C) as well as TPEN (Figures 6C,D). These results strongly support mitochondrial ROS generation as a sequela of mitochondrial Zn^2+^ accumulation.

In summary, this study shows that TRPM2-dependent dynamic alteration in the intracellular Zn^2+^ homeostasis and lysosomal and mitochondrial dysfunctions are important in contributing to ROS-induced hippocampal neuronal death. These novel insights should be useful in facilitating better understanding ROS-induced neuronal death implicated in ischemia-reperfusion brain damage.

## Author Contributions

L-HJ conceived the research. L-HJ, XL and WY designed the experiments. XL performed experiments and analyzed data, and WY carried out initial experiments and contributed to discussion. L-HJ and XL wrote the manuscript.

## Conflict of Interest Statement

The authors declare that the research was conducted in the absence of any commercial or financial relationships that could be construed as a potential conflict of interest.

## References

[B1] Abuarab N. Munsey T. S. Jiang L. H. Li J. Sivaprasadarao A. (2017). High glucose-induced ROS activates TRPM2 to trigger lysosomal membrane permeabilization and Zn^2+^-mediated mitochondrial fission. Sci. Signal. 10:eaal4161. 10.1126/scisignal.aal4161 28765513

[B2] Alano C. C. Garnier P. Ying W. H. Higashi Y. Kauppinen T. M. Swanson R. A. (2010). NAD^+^ depletion is necessary and sufficient for poly(ADP-ribose) polymerase-1-mediated neuronal death. J. Neurosci. 30, 2967–2978. 10.1523/JNEUROSCI.5552-09.2010 20181594 PMC2864043

[B3] Alim I. Teves L. Li R. Mori Y. Tymianski M. (2013). Modulation of NMDAR subunit expression by TRPM2 channels regulates neuronal vulnerability to ischemic cell death. J. Neurosci. 33, 17264–17277. 10.1523/JNEUROSCI.1729-13.2013 24174660 PMC6618359

[B4] Bao L. Chen S. J. Conrad K. Keefer K. Abraham T. Lee J. P.. (2016). Depletion of the human ion channel TRPM2 in neuroblastoma demonstrates its key role in cell survival through modulation of mitochondrial reactive oxygen species and bioenergetics. J. Biol. Chem. 291, 24449–24464. 10.1074/jbc.M116.747147 27694440 PMC5114400

[B5] Beaudoin G. M. III. Lee S. H. Singh D. Yuan Y. Ng Y. G. Reichardt L. F.. (2012). Culturing pyramidal neurons from the early postnatal mouse hippocampus and cortex. Nat. Protoc. 7, 1741–1754. 10.1038/nprot.2012.099 22936216

[B6] Chan P. H. (2001). Reactive oxygen radicals in signaling and damage in the ischemic brain. J. Cereb. Blood Flow Metab. 21, 2–14. 10.1097/00004647-200101000-00002 11149664

[B7] Chen H. Yoshioka H. Kim G. S. Jung J. E. Okami N. Sakata H.. (2011). Oxidative stress in ischemic brain damage: mechanisms of cell death and potential molecular targets for neuroprotection. Antioxid. Redox Signal. 14, 1505–1517. 10.1089/ars.2010.3576 20812869 PMC3061196

[B8] Cole K. K. Perez-Polo J. R. (2002). Poly(ADP-ribose) polymerase inhibition prevents both apoptotic-like delayed neuronal death and necrosis after H_2_O_2_ injury. J. Neurochem. 82, 19–29. 10.1046/j.1471-4159.2002.00935.x 12091461

[B9] Colvin R. A. Fontaine C. P. Laskowski M. Thomas D. (2003). Zn^2+^ transporters and Zn^2+^ homeostasis in neurons. Eur. J. Pharmacol. 479, 171–185. 10.1016/j.ejphar.2003.08.067 14612148

[B10] Dantzer F. Amé J. C. Schreiber V. Nakamura J. Menissier-de Murcia J. de Murcia G. (2006). Poly(ADP-ribose) polymerase-1 activation during DNA damage and repair. Meth. Enzymol. 409, 493–510. 10.1016/s0076-6879(05)09029-4 16793420

[B11] De Vos K. J. Allan V. J. Grierson A. J. Sheetz M. P. (2005). Mitochondrial function and actin regulate dynamin-related protein 1-dependent mitochondrial fission. Curr. Biol. 15, 678–683. 10.1016/j.cub.2005.02.064 15823542

[B12] Dineley K. E. Richards L. L. Votyakova T. V. Reynolds I. J. (2005). Zinc causes loss of membrane potential and elevates reactive oxygen species in rat brain mitochondria. Mitochondrion 5, 55–65. 10.1016/j.mito.2004.11.001 16060292

[B13] Dineley K. E. Votyakova T. V. Reynolds I. J. (2003). Zinc inhibition of cellular energy production: implications for mitochondria and neurodegeneration. J. Neurochem. 85, 563–570. 10.1046/j.1471-4159.2003.01678.x 12694382

[B14] Doyle K. P. Simon R. P. Stenzel-Poore M. P. (2008). Mechanisms of ischemic brain damage. Neuropharmacology 55, 310–318. 10.1016/j.neuropharm.2008.01.005 18308346 PMC2603601

[B15] Du J. Xie J. Yue L. (2009). Intracellular calcium activates TRPM2 and its alternative spliced isoforms. Proc. Natl. Acad. Sci. U S A 106, 7239–7244. 10.1073/pnas.0811725106 19372375 PMC2678461

[B16] Dunn K. W. Kamocka M. M. McDonald J. H. (2011). A practical guide to evaluating colocalization in biological microscopy. Am. J. Physiol. Cell Physiol. 300, C723–C742. 10.1152/ajpcell.00462.2010 21209361 PMC3074624

[B18] Fonfria E. Marshall I. C. Boyfield I. Skaper S. D. Hughes J. P. Owen D. E.. (2005). Amyloid β-peptide(1–42) and hydrogen peroxide-induced toxicity are mediated by TRPM2 in rat primary striatal cultures. J. Neurochem. 95, 715–723. 10.1111/j.1471-4159.2005.03396.x 16104849

[B19] Fonfria E. Mattei C. Hill K. Brown J. T. Randall A. Benham C. D.. (2006). TRPM2 is elevated in the tMCAO stroke model, transcriptionally regulated and functionally expressed in C13 microglia. J. Recept. Sig. Transd. 26, 179–198. 10.1080/10799890600637522 16777714

[B20] Gao G. F. Wang W. W. Tadagavadi R. K. Briley N. E. Love M. I. Miller B. A.. (2014). TRPM2 mediates ischemic kidney injury and oxidant stress through RAC1. J. Clin. Invest. 124, 4989–5001. 10.1172/JCI76042 25295536 PMC4347231

[B21] Gee K. R. Zhou Z. L. Ton-That D. Sensi S. L. Weiss J. H. (2002). Measuring zinc in living cells. A new generation of sensitive and selective fluorescent probes. Cell Calcium 31, 245–251. 10.1016/S0143-4160(02)00053-2 12098227

[B22] Hara Y. Wakamori M. Ishii M. Maeno E. Nishida M. Yoshida T.. (2002). LTRPC2 Ca^2+^-permeable channel activated by changes in redox status confers susceptibility to cell death. Mol. Cell 9, 163–173. 10.1016/s1097-2765(01)00438-5 11804595

[B23] Hwang J. J. Lee S. J. Kim T. Y. Cho J. H. Koh J. Y. (2008). Zinc and 4-hydroxy-2-nonenal mediate lysosomal membrane permeabilization induced by H2O2 in cultured hippocampal neurons. J. Neurosci. 28, 3114–3122. 10.1523/JNEUROSCI.0199-08.2008 18354014 PMC6670692

[B24] Jia J. Verma S. Nakayama S. Quillinan N. Grafe M. R. Hurn P. D.. (2011). Sex differences in neuroprotection provided by inhibition of TRPM2 channels following experimental stroke. J. Cereb. Blood Flow Metab. 31, 2160–2168. 10.1038/jcbfm.2011.77 21587268 PMC3210342

[B27] Jiang Q. Gao Y. Wang C. Tao R. Wu Y. Zhan K.. (2017). Nitration of TRPM2 as a molecular switch induces autophagy during brainp injury. Antioxid. Redox Signal. 27, 1297–1316. 10.1089/ars.2016.6873 28292196

[B25] Jiang D. Sullivan P. G. Sensi S. L. Steward O. Weiss J. H. (2001). Zn^2+^ induces permeability transition pore opening and release of pro-apoptotic peptides from neuronal mitochondria. J. Biol. Chem. 276, 47524–47529. 10.1074/jbc.M108834200 11595748

[B26] Jiang L. H. Yang W. Zou J. Beech D. J. (2010). TRPM2 channel properties, functions and therapeutic potentials. Expert Opin. Ther. Targets 14, 973–988. 10.1517/14728222.2010.510135 20670202

[B28] Kalyanaraman B. (2013). Teaching the basics of redox biology to medical and graduate students: oxidants, antioxidants and disease mechanisms. Redox Biol. 1, 244–257. 10.1016/j.redox.2013.01.014 24024158 PMC3757692

[B29] Kaneko S. Kawakami S. Hara Y. Wakamori M. Itoh E. Minami T.. (2006). A critical role of TRPM2 in neuronal cell death by hydrogen peroxide. J. Pharmacol. Sci. 101, 66–76. 10.1254/jphs.fp0060128 16651700

[B30] Kheradpezhouh E. Ma L. Morphett A. Barritt G. J. Rychkov G. Y. (2014). TRPM2 channels mediate acetaminophen-induced liver damage. Proc. Natl. Acad. Sci. U S A 111, 3176–3181. 10.1073/pnas.1322657111 24569808 PMC3939869

[B31] Kitagawa K. Matsumoto M. Oda T. Niinobe M. Hata R. Handa N.. (1990). Free radical generation during brief period of cerebral ischemia may trigger delayed neuronal death. Neuroscience 35, 551–558. 10.1016/0306-4522(90)90328-2 2199842

[B32] Koopman W. J. Verkaart S. Visch H. J. van der Westhuizen F. H. Murphy M. P. van den Heuvel L. W.. (2005). Inhibition of complex I of the electron transport chain causes O_2_^−.^-mediated mitochondrial outgrowth. Am. J. Physiol. Cell Physiol. 288, C1440–C1450. 10.1152/ajpcell.00607.2004 15647387

[B33] Kraft R. Grimm C. Grosse K. Hoffmann A. Sauerbruch S. Kettenmann H.. (2004). Hydrogen peroxide and ADP-ribose induce TRPM2-mediated calcium influx and cation currents in microglia. Am. J. Physiol. Cell Physiol. 286, C129–C137. 10.1152/ajpcell.00331.2003 14512294

[B34] Lange I. Yamamoto S. Partida-Sanchez S. Mori Y. Fleig A. Penner R. (2009). TRPM2 functions as a lysosomal Ca^2+^-release channel in β cells. Sci. Signal. 2:ra23. 10.1126/scisignal.2000278 19454650 PMC2779714

[B35] Lee J. Y. Kim J. H. Palmiter R. D. Koh J. Y. (2003). Zinc released from metallothionein-iii may contribute to hippocampal CA1 and thalamic neuronal death following acute brain injury. Exp. Neurol. 184, 337–347. 10.1016/s0014-4886(03)00382-0 14637104

[B36] Li C. Meng L. Li X. Li D. Jiang L. H. (2015). Non-NMDAR neuronal Ca^2+^-permeable channels in delayed neuronal death and as potential therapeutic targets for ischemic brain damage. Expert Opin. Ther. Targets 19, 879–892. 10.1517/14728222.2015.1021781 25732672

[B37] Li F. Munsey T. S. Sivaprasadarao A. (2017). TRPM2-mediated rise in mitochondrial Zn^2+^ promotes palmitate-induced mitochondrial fission and pancreatic β-cell death in rodents. Cell Death Diff. 24, 1999–2012. 10.1038/cdd.2017.118 28753206 PMC5686341

[B38] Lo E. H. Dalkara T. Moskowitz M. A. (2003). Mechanisms, challenges and opportunities in stroke. Nat. Rev. Neurosci. 4, 399–415. 10.1038/nrn1106 12728267

[B39] Manna P. T. Munsey T. S. Abuarab N. Li F. Asipu A. Howell G.. (2015). TRPM2-mediated intracellular Zn^2+^ release triggers pancreatic β-cell death. Biochem. J. 466, 537–546. 10.1042/BJ20140747 25562606

[B40] McCord J. M. (1985). Oxygen-derived free radicals in postischemic tissue injury. N. Engl. J. Med. 312, 159–163. 10.1056/nejm198501173120305 2981404

[B41] Medvedeva Y. V. Lin B. Shuttleworth C. W. Weiss J. H. (2009). Intracellular Zn^2+^ accumulation contributes to synaptic failure, mitochondrial depolarization, and cell death in an acute slice oxygen-glucose deprivation model of ischemia. J. Neurosci. 29, 1105–1114. 10.1523/JNEUROSCI.4604-08.2009 19176819 PMC2637403

[B42] Mortadza S. S. Sim J. A. Stacey M. Jiang L. H. (2017). Signalling mechanisms mediating Zn^2+^-induced TRPM2 channel activation and cell death in microglial cells. Sci. Rep. 7:45032. 10.1038/srep45032 28322340 PMC5359577

[B43] Nakayama S. Vest R. Traystman R. J. Herson P. S. (2013). Sexually dimorphic response of TRPM2 inhibition following cardiac arrest-induced global cerebral ischemia in mice. J. Mol. Neurosci. 51, 92–98. 10.1007/s12031-013-0005-9 23532768 PMC3728170

[B44] Olah M. E. Jackson M. F. Li H. Perez Y. Sun H. S. Kiyonaka S.. (2009). Ca^2+^-dependent induction of TRPM2 currents in hippocampal neurons. J. Physiol. 587, 965–979. 10.1113/jphysiol.2008.162289 19124544 PMC2673769

[B45] Ostapchenko V. G. Chen M. A. Guzman M. S. Xie Y. F. Lavine N. Fan J.. (2015). The transient receptor potential melastatin 2 (TRPM2) channel contributes to β-amyloid oligomer-related neurotoxicity and memory impairment. J. Neurosci. 35, 15157–15169. 10.1523/JNEUROSCI.4081-14.2015 26558786 PMC6605355

[B46] Park L. Wang G. Moore J. Girouard H. Zhou P. Anrather J.. (2014). The key role of transient receptor potential melastatin-2 channels in amyloid-β-induced neurovascular dysfunction. Nat. Commun. 5:5318. 10.1038/ncomms6318 25351853 PMC4283829

[B47] Perraud A. L. Fleig A. Dunn C. A. Bagley L. A. Launay P. Schmitz C.. (2001). ADP-ribose gating of the calcium-permeable LTRPC2 channel revealed by Nudix motif homology. Nature 411, 595–599. 10.1038/35079100 11385575

[B48] Sanderson T. H. Reynolds C. A. Kumar R. Przyklenk K. Hüttemann M. (2013). Molecular mechanisms of ischemia-reperfusion injury in brain: pivotal role of the mitochondrial membrane potential in reactive oxygen species generation. Mol. Neurobiol. 47, 9–23. 10.1007/s12035-012-8344-z 23011809 PMC3725766

[B49] Sano Y. Inamura K. Miyake A. Mochizuki S. Yokoi H. Matsushime H.. (2001). Immunocyte Ca^2+^ influx system mediated by LTRPC2. Science 293, 1327–1330. 10.1126/science.1062473 11509734

[B50] Sensi S. L. Paoletti P. Koh J.-Y. Aizenman E. Bush A. I. Hershfinkel M. (2011). The neurophysiology and pathology of brain zinc. J. Neurosci. 31, 16076–16085. 10.1523/jneurosci.3454-11.2011 22072659 PMC3223736

[B51] Sensi S. L. Ton-That D. Sullivan P. G. Jonas E. A. Gee K. R. Kaczmarek L. K.. (2003). Modulation of mitochondrial function by endogenous Zn^2+^ pools. Proc. Natl. Acad. Sci. U S A 100, 6157–6162. 10.1073/pnas.1031598100 12724524 PMC156342

[B52] Shimizu T. Dietz R. M. Cruz-Torres I. Strnad F. Garske A. K. Moreno M.. (2016). Extended therapeutic window of a novel peptide inhibitor of TRPM2 channels following focal cerebral ischemia. Exp. Neurol. 283, 151–156. 10.1016/j.expneurol.2016.06.015 27317297 PMC5240152

[B53] Shimizu T. Macey T. A. Quillinan N. Klawitter J. Perraud A. L. Traystman R. J.. (2013). Androgen and PARP-1 regulation of TRPM2 channels after ischemic injury. J. Cereb. Blood Flow Metab. 33, 1549–1555. 10.1038/jcbfm.2013.105 23801245 PMC3790922

[B54] Shuttleworth C. W. Weiss J. H. (2011). Zinc: new clues to diverse roles in brain ischemia. Trends Pharmacol. Sci. 32, 480–486. 10.1016/j.tips.2011.04.001 21621864 PMC3148334

[B55] Sun Y. Sukumaran P. Selvaraj S. Cilz N. I. Schaar A. Lei S.. (2016). TRPM2 promotes neurotoxin MPP^+^/MPTP-induced cell death. Mol. Neurobiol. [Epub ahead of print]. 10.1007/s12035-016-0338-9 27957685 PMC5468501

[B56] Togashi K. Hara Y. Tominaga T. Higashi T. Konishi Y. Mori Y.. (2006). TRPM2 activation by cyclic ADP-ribose at body temperature is involved in insulin secretion. EMBO J. 25, 1804–1815. 10.1038/sj.emboj.7601083 16601673 PMC1456947

[B57] Tóth B. Csanády L. (2010). Identification of direct and indirect effectors of the transient receptor potential melastatin 2 (TRPM2) cation channel. J. Biol. Chem. 285, 30091–30102. 10.1074/jbc.m109.066464 20650899 PMC2943302

[B58] Uttara B. Singh A. V. Zamboni P. Mahajan R. T. (2009). Oxidative stress and neurodegenerative diseases: a review of upstream and downstream antioxidant therapeutic options. Curr. Neuropharmacol. 7, 65–74. 10.2174/157015909787602823 19721819 PMC2724665

[B59] Verma S. Quillinan N. Yang Y.-F. Nakayama S. Cheng J. Kelley M. H.. (2012). TRPM2 channel activation following *in vitro* ischemia contributes to male hippocampal cell death. Neurosci. Lett. 530, 41–46. 10.1016/j.neulet.2012.09.044 23041043 PMC3518922

[B60] Virág L. Robaszkiewicz A. Rodriguez-Vargas J. M. Oliver F. J. (2013). Poly(ADP-ribose) signaling in cell death. Mol. Aspects Med. 34, 1153–1167. 10.1016/j.mam.2013.01.007 23416893

[B61] Xia R. Mei Z.-Z. Mao H.-J. Yang W. Dong L. Bradley H.. (2008). Identification of pore residues engaged in determining divalent cationic permeation in transient receptor potential melastatin subtype channel 2. J. Biol. Chem. 283, 27426–27432. 10.1074/jbc.m801049200 18687688 PMC2562080

[B62] Xu S. Zhang R. Niu J. Cui D. Xie B. Zhang B.. (2012). Oxidative stress mediated-alterations of the microRNA expression profile in mouse hippocampal neurons. Int. J. Mol. Sci. 13, 16945–16960. 10.3390/ijms131216945 23443129 PMC3546732

[B63] Yang W. Manna P. T. Zou J. Luo J. Beech D. J. Sivaprasadarao A.. (2011). Zinc inactivates melastatin transient receptor potential 2 channels via the outer pore. J. Biol. Chem. 286, 23789–23798. 10.1074/jbc.M111.247478 21602277 PMC3129160

[B64] Ye M. Yang W. Ainscough J. F. Hu X.-P. Li X. Sedo A.. (2014). TRPM2 channel deficiency prevents delayed cytosolic Zn^2+^ accumulation and CA1 pyramidal neuronal death after transient global ischemia. Cell Death Dis. 5:e1541. 10.1038/cddis.2014.494 25429618 PMC4260752

[B65] Zou J. Ainscough J. F. Yang W. Sedo A. Yu S.-P. Mei Z. Z.. (2013). A differential role of macrophage TRPM2 channels in Ca^2+^ signaling and cell death in early responses to H_2_O_2_. Am. J. Physiol. Cell Physiol. 305, C61–C69. 10.1152/ajpcell.00390.2012 23596170

