# Supplementary material for: Alteration in Intracellular Zn2+ Homeostasis as a Result of TRPM2 Channel Activation Contributes to ROS-Induced Hippocampal Neuronal Death
Source: Front Mol Neurosci. 2017 Dec 12;10:414. doi: 10.3389/fnmol.2017.00414 (PMC5732979; doi:10.3389/fnmol.2017.00414)

Supplementary data by Xin Li et al

Figure 1 H_2O_2-induced concentration- and duration-dependent hippocampal neuronal death. Representative fluorescence images showing PI and Hoechst staining of WT neurons under control (CTL) or treatment with 30-300 μM H_2O_2 for 2, 8 and 24 hr. Scale bar is 100 μm. The mean data are shown in Fig. 1a.

Figure 2 Lack of an inhibition of PJ34 on H_2O_2-induced neuronal death in TRPM2-KO neurons. Mean percentage of PI positive TRPM2-KO hippocampal neurons under control (CTL) or neurons exposed to 300 μM H_2O_2 for 24 hr or treated with 1 μM PJ34, 30 min prior to and during exposure to 300 μM H_2O_2, from 3 independent experiments with each independent experiment examining 450-600 neurons. *** $p < 0.005$ indicates difference from CTL. NS, no significant difference. The black bar shows H_2O_2-induced neuronal death in WT neurons as shown in Fig. 1c.

Figure 3 No effect of PJ34 and 2-APB on the $[Zn^{2+}]_c$ in H_2O_2-exposed TRPM2-KO neurons. (**a**) Representative fluorescence images showing FluoZin3 fluorescence in TRPM2-KO hippocampal neurons under control (CTL) or exposed to 300 μM H_2O_2 for 30 min or treated with 1 μM PJ34 or 10 μM 2-APB, 30 min prior to and during exposure to H_2O_2. The scale bar is 10 μm. (**b**) Mean FluoZin3 fluorescence intensity under indicated conditions from 3 independent experiments with a total of 40-60 neurons examined. All values are normalized to the basal fluorescence level in control neurons in matched experiments. NS, no significant difference.

Figure 4 Effect of exposure to H_2O_2 on TRPM2 protein expression. Representative immunofluorescent images showing no increase in the TRPM2 expression in WT neurons under control (CTL) conditions or after exposure to 100 μM or 300 μM H_2O_2 for 30 min. Similar observations were made in two independent experiments. Scale bar is 20 μm.

Supplementary Fig. 1

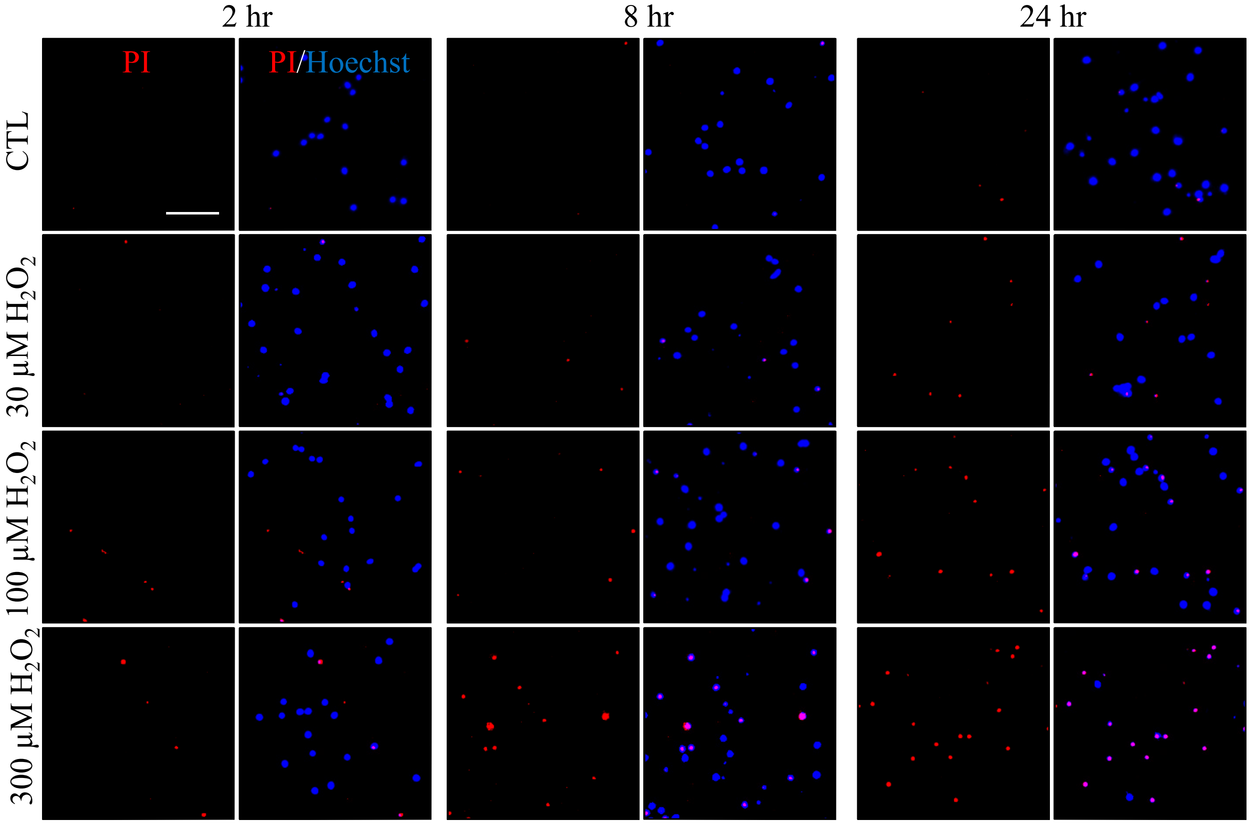

Supplementary Fig. 2

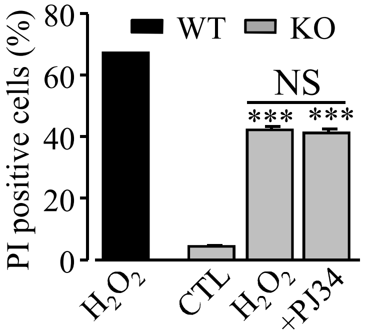

Supplementary Fig. 3

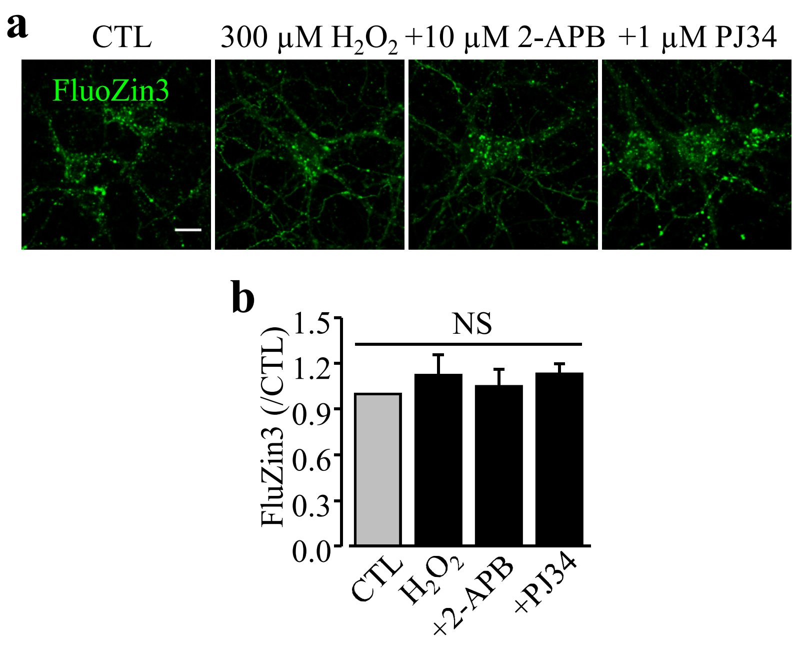

Supplementary Fig. 4

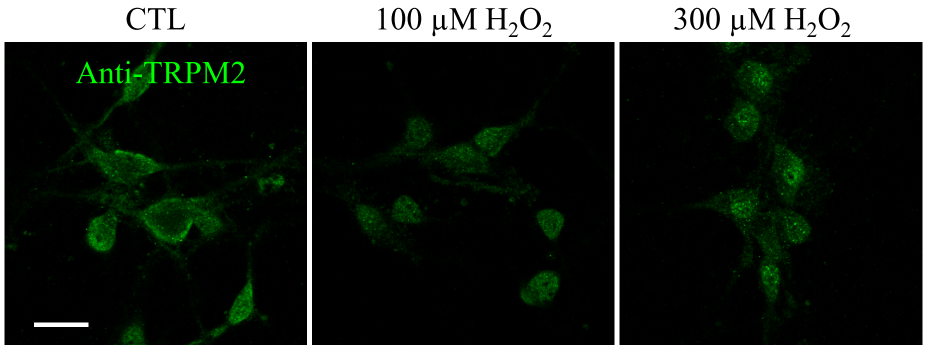

Supplement: Supplementary file 1 [file Data_Sheet_1.pdf]
